# Supplementary material for: Genetic Variation and Metapopulation Structure Inform Recovery Goals in a Threatened Species
Source: Genes (Basel). 2025 Jun 8;16(6):694. doi: 10.3390/genes16060694 (PMC12192496; doi:10.3390/genes16060694)
Supplement: Supplementary file 1 [file genes-16-00694-s001.zip › genes-3640727-supplementary.pdf]

Genetic variation and metapopulation structure inform recovery goals in a threatened species

SUPPLEMENTARY FIGURES

Figure S1: PC axes 3 and 4 for all, neutral, and adaptive SNPs

Figure S2: Evanno and likelihood plots for identifying the best K for STRUCTURE for all, neutral, and adaptive SNPs

Figure S3: STRUCTURE bar plots with all SNPs

Figure S4: STRUCTURE bar plots with neutral SNPs

Figure S5: STRUCTURE bar plots with adaptive SNPs

Figure S6: PCA outputs with site Round Valley

Figure S7: Pairwise F_{ST} with all SNPs and adaptive SNPs

SUPPLEMENTARY TABLES

Table S1: Mantel test metrics

Table S2: Private alleles per ESU per year

Table S3: Pairwise F_{ST} confidence intervals with neutral SNPs

Table S4: Private alleles per MU per year

FIGURE S1: PC axes 3 and 4 for (a) all loci, (b) neutral loci, and (c) adaptive loci. Circle points denote the sample was collected in 2016 and triangles for 2020. Colors represent each site.

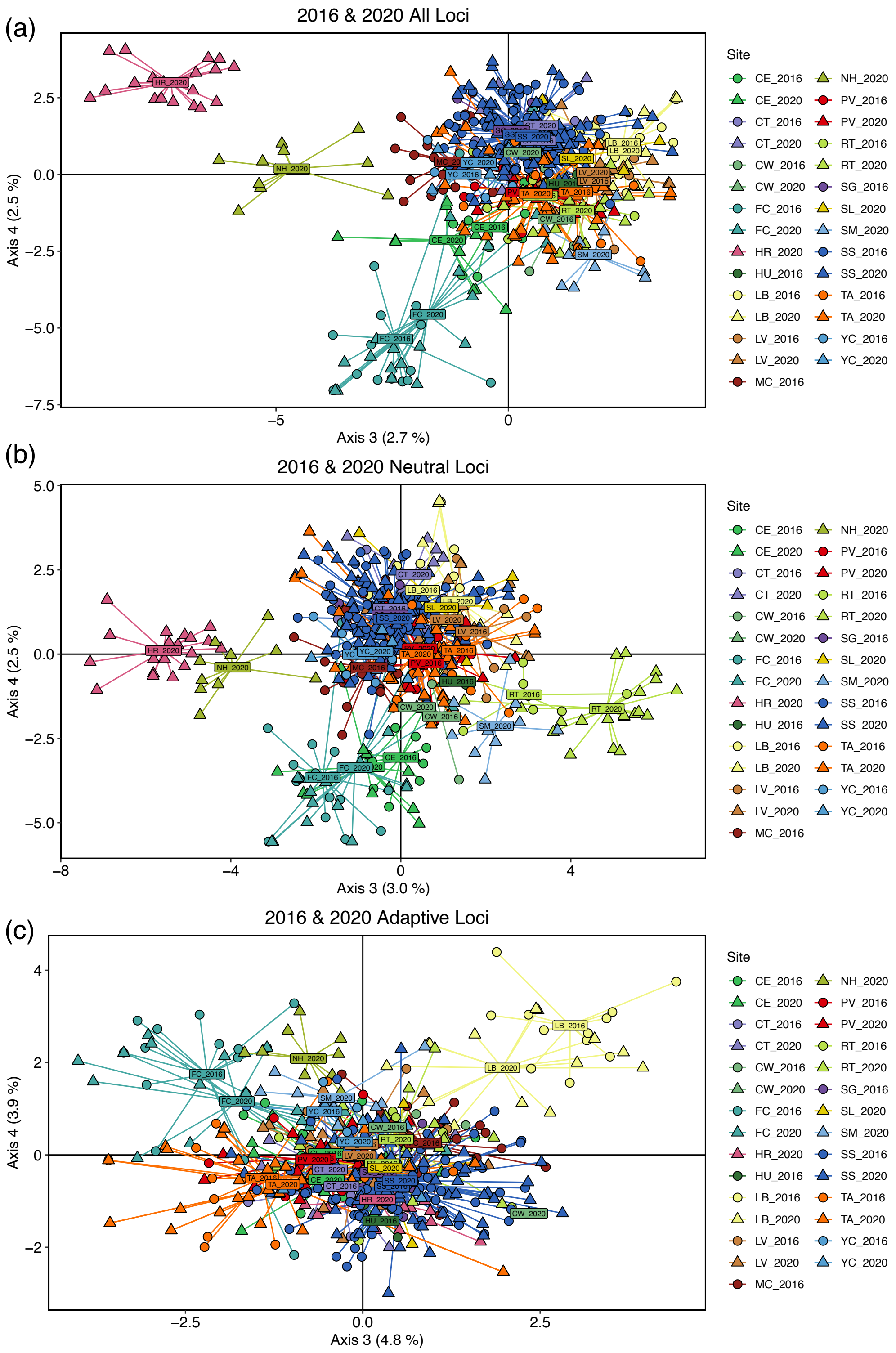

FIGURE S2: Evaluating the best-fit K using the Evanno method for (1) all SNPs, (2) neutral SNPs, and (3) adaptive SNPs. (A) Mean $L(K)$ over 10 runs for each K value. (B) Rate of change of the likelihood distribution calculated as $L'K = L(K) - L(K-1)$. (C) Absolute values of the second order rate of change of the likelihood distribution calculated according to: $|L''K| = |L'(K+1) - L'(K)$. (D) ΔK calculated as $\Delta K = m|L''(K)|/s(L(K)]$.

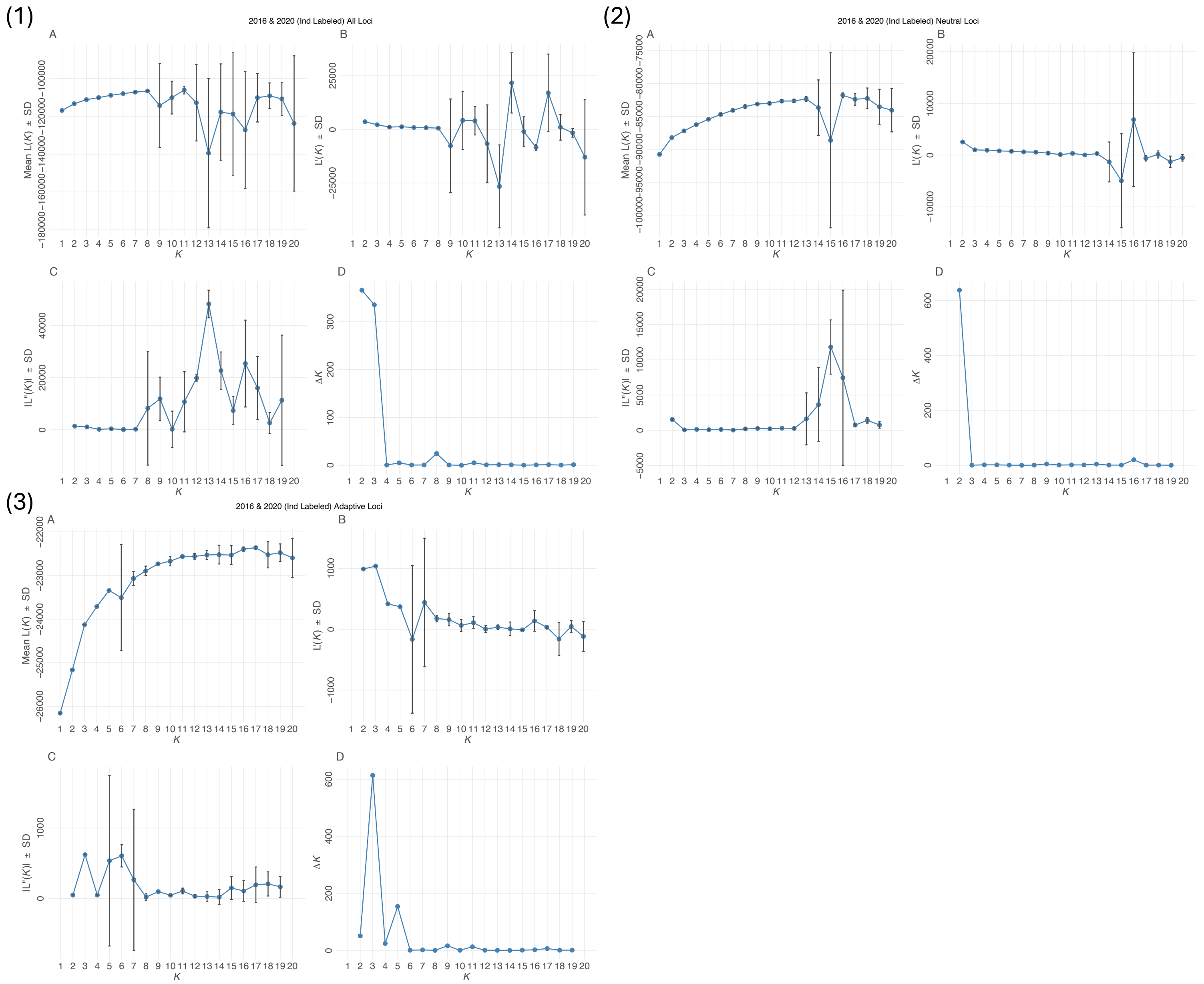

FIGURE S3a: K= 2-4 STRUCTURE bar plots with all loci displaying inferred clustering and individual ancestry estimates of NIDGS. Each color represents a distinct genetic cluster, each vertical bar represents the portion of ancestry of a single individual to the different genetic cluster. Individuals are grouped into populations which are ordered geographically from west to east.

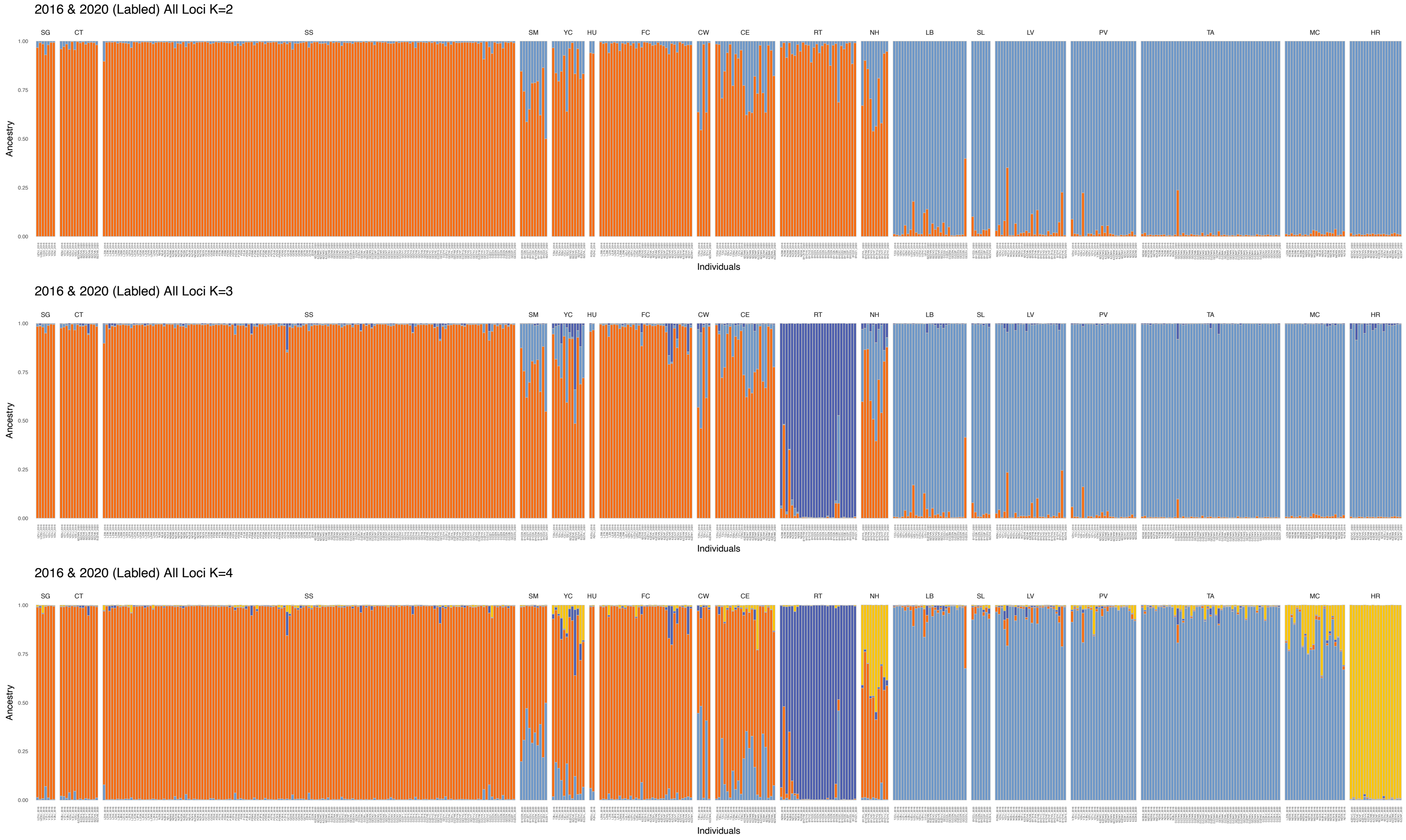

FIGURE S3b: K= 5-7 STRUCTURE bar plots with all loci displaying inferred clustering and individual ancestry estimates of NIDGS. Each color represents a distinct genetic cluster, each vertical bar represents the portion of ancestry of a single individual to the different genetic cluster. Individuals are grouped into populations which are ordered geographically from west to east.

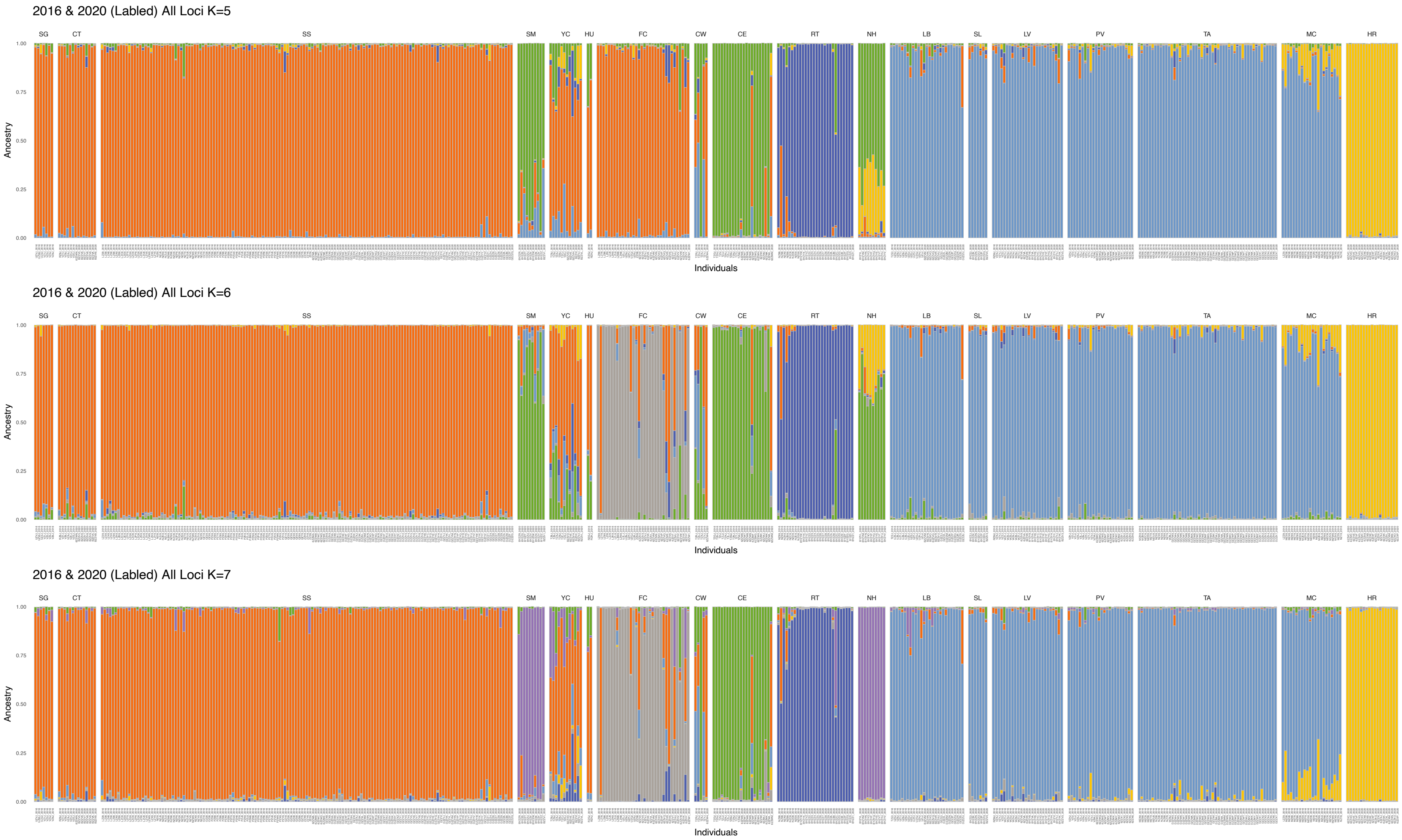

FIGURE S3c: K= 8-10 STRUCTURE bar plots with all loci displaying inferred clustering and individual ancestry estimates of NIDGS. Each color represents a distinct genetic cluster, each vertical bar represents the portion of ancestry of a single individual to the different genetic cluster. Individuals are grouped into populations which are ordered geographically from west to east.

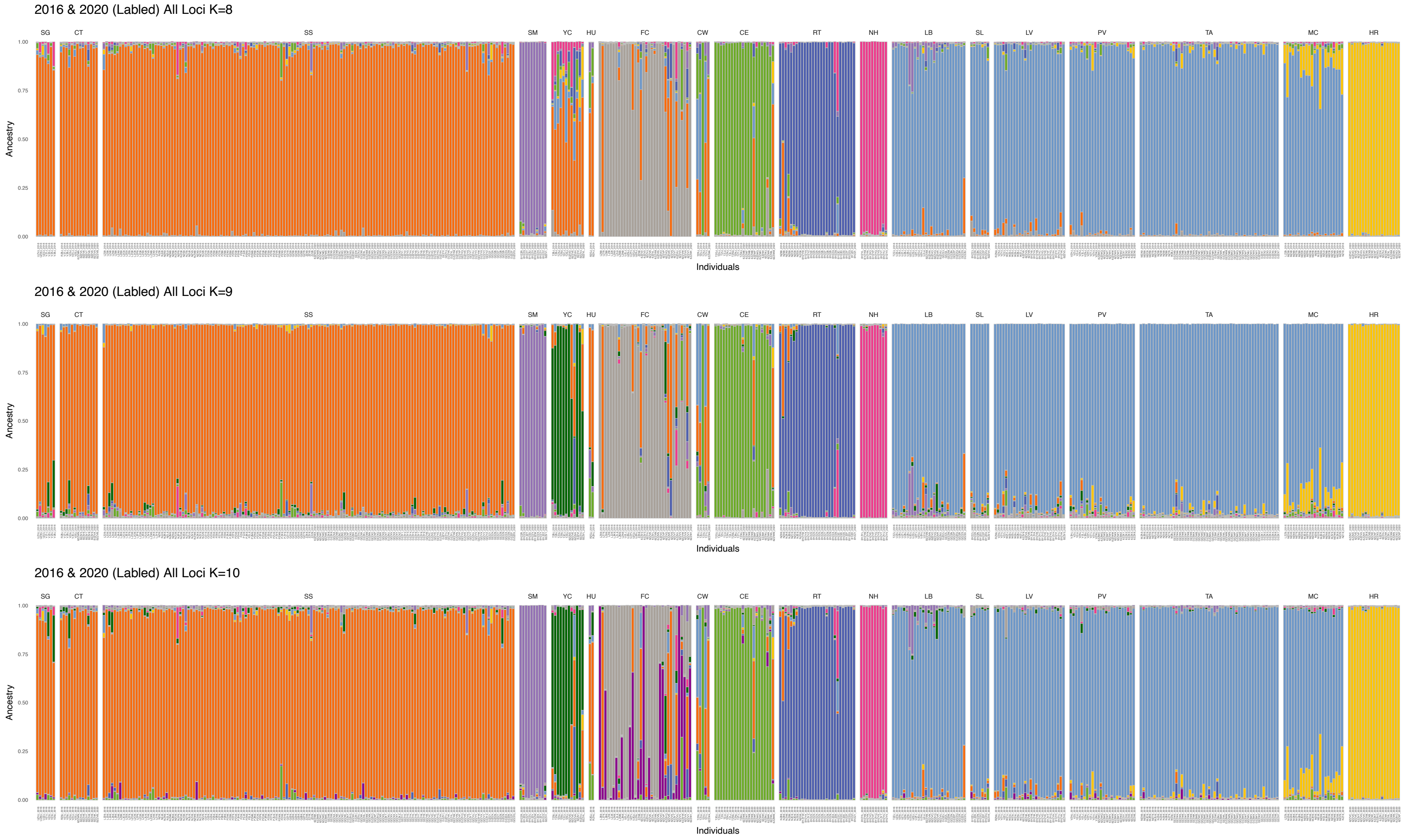

FIGURE S4a: K= 2-4 STRUCTURE bar plots with neutral loci displaying inferred clustering and individual ancestry estimates of NIDGS. Each color represents a distinct genetic cluster, each vertical bar represents the portion of ancestry of a single individual to the different genetic cluster. Individuals are grouped into populations which are ordered geographically from west to east.

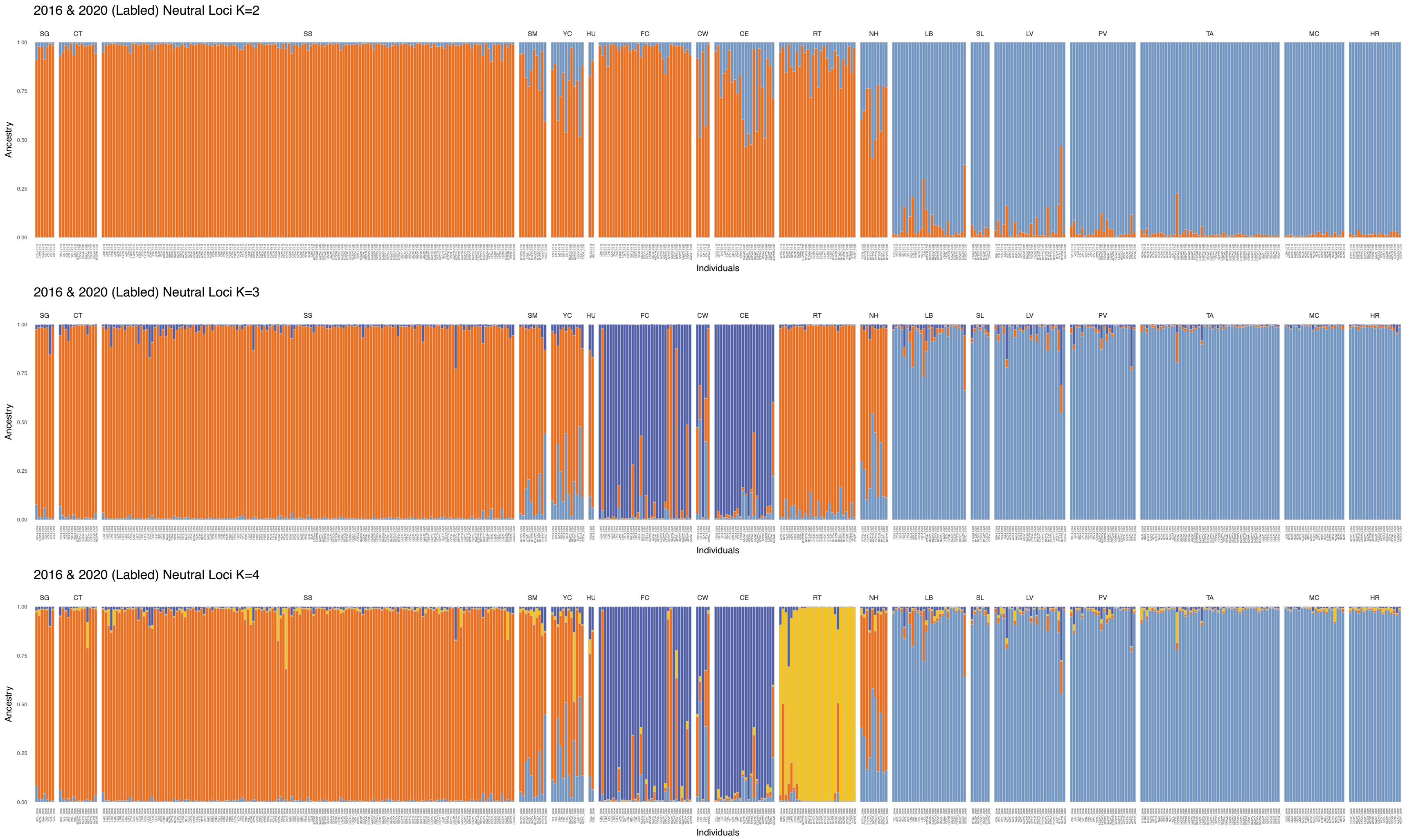

FIGURE S4b: K= 5-7 STRUCTURE bar plots with neutral loci displaying inferred clustering and individual ancestry estimates of NIDGS. Each color represents a distinct genetic cluster, each vertical bar represents the portion of ancestry of a single individual to the different genetic cluster. Individuals are grouped into populations which are ordered geographically from west to east.

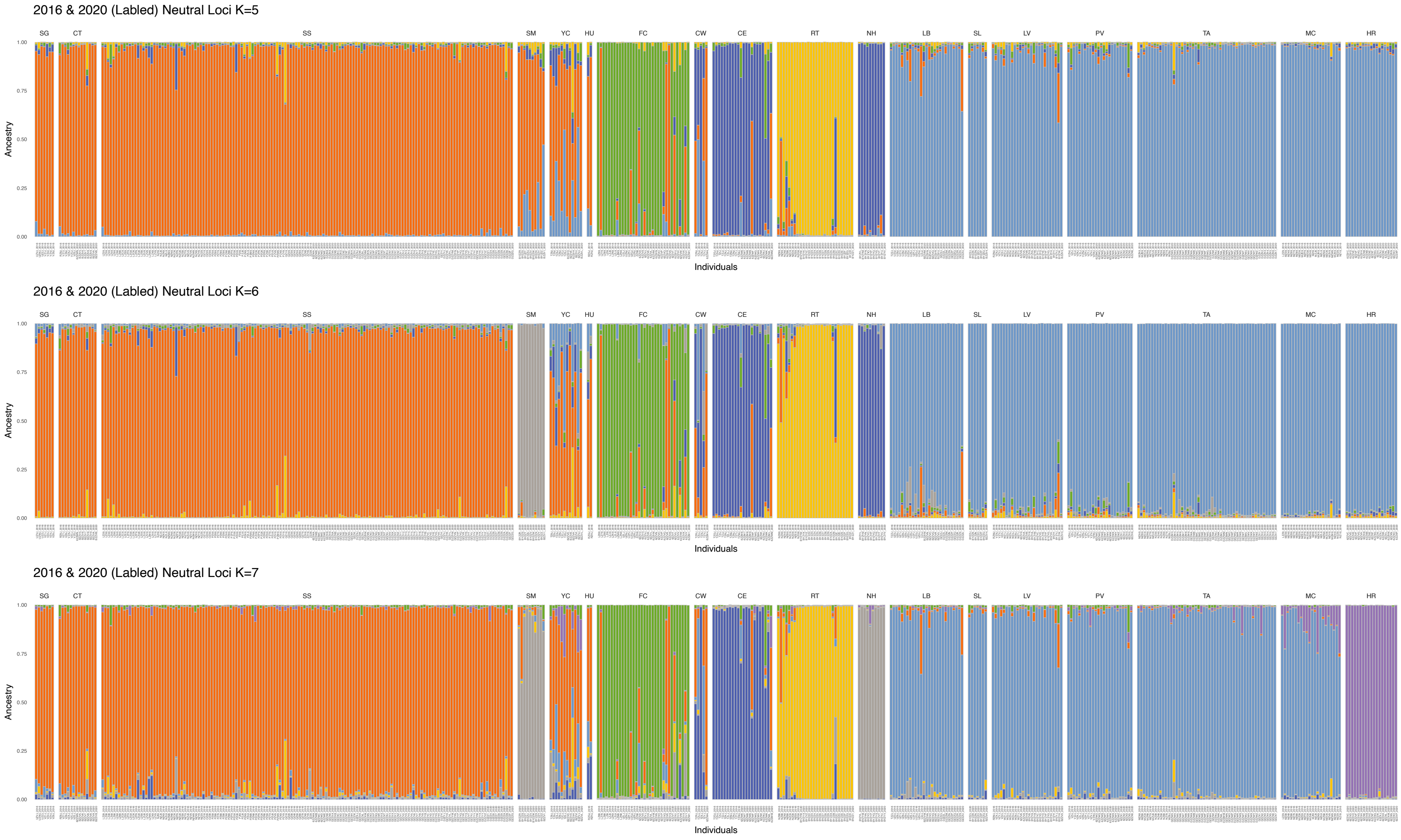

FIGURE S4c: K= 8-10 STRUCTURE bar plots with neutral loci displaying inferred clustering and individual ancestry estimates of NIDGS. Each color represents a distinct genetic cluster, each vertical bar represents the portion of ancestry of a single individual to the different genetic cluster. Individuals are grouped into populations which are ordered geographically from west to east.

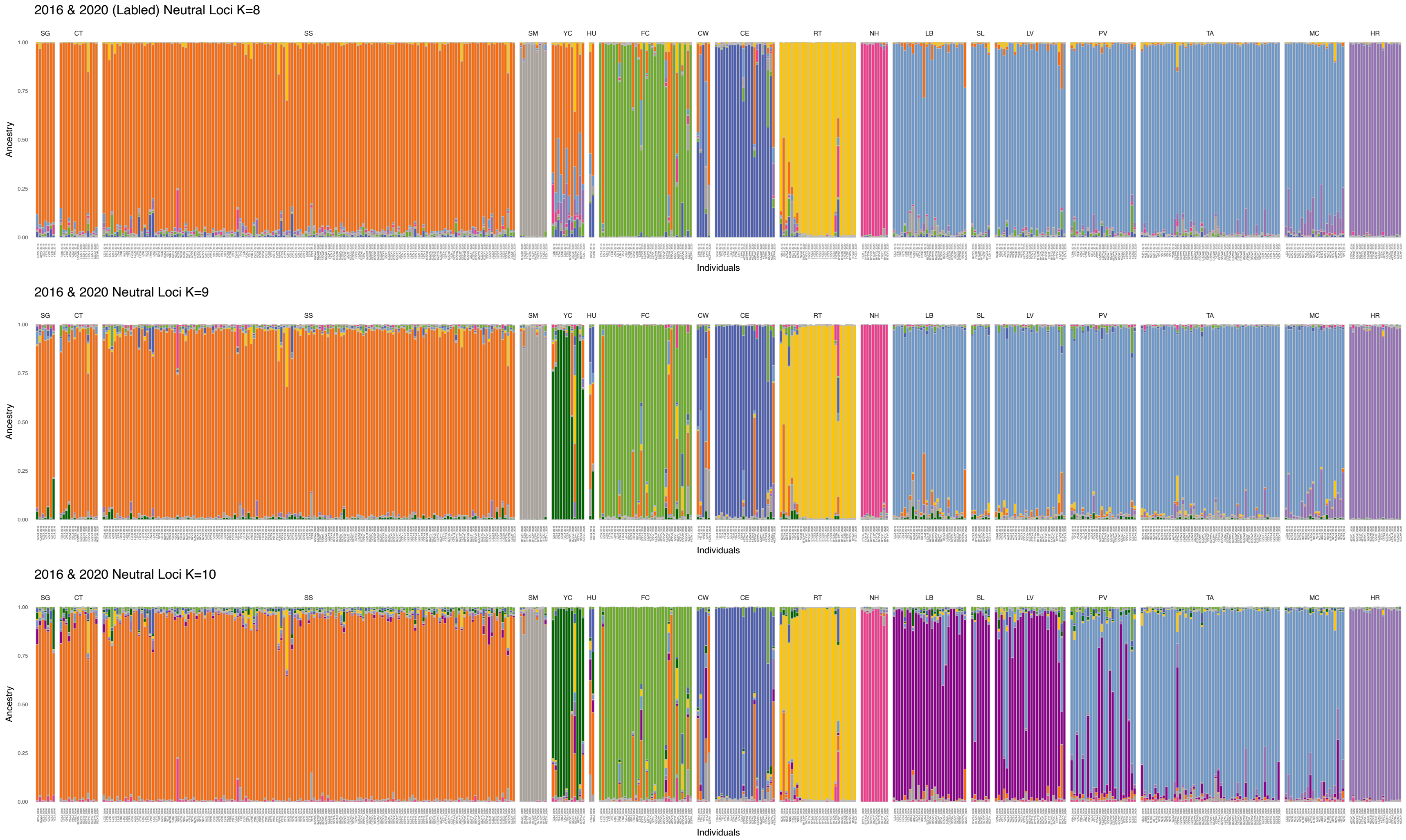

FIGURE S5a: K= 2-4 STRUCTURE bar plots with adaptive loci displaying inferred clustering and individual ancestry estimates of NIDGS. Each color represents a distinct genetic cluster, each vertical bar represents the portion of ancestry of a single individual to the different genetic cluster. Individuals are grouped into populations which are ordered geographically from west to east.

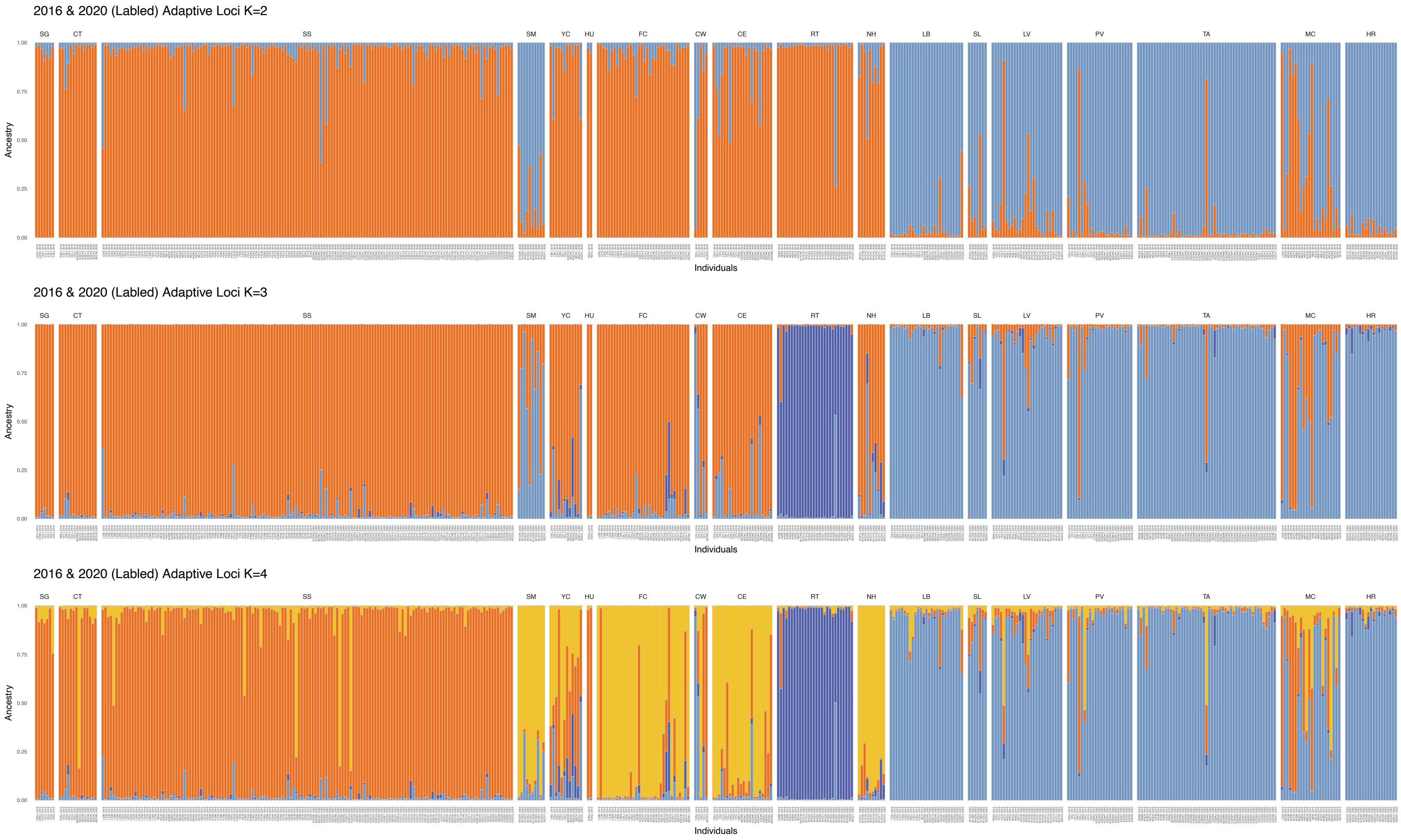

FIGURE S5b: K= 5-8 STRUCTURE bar plots with adaptive loci displaying inferred clustering and individual ancestry estimates of NIDGS. Each color represents a distinct genetic cluster, each vertical bar represents the portion of ancestry of a single individual to the different genetic cluster. Individuals are grouped into populations which are ordered geographically from west to east.

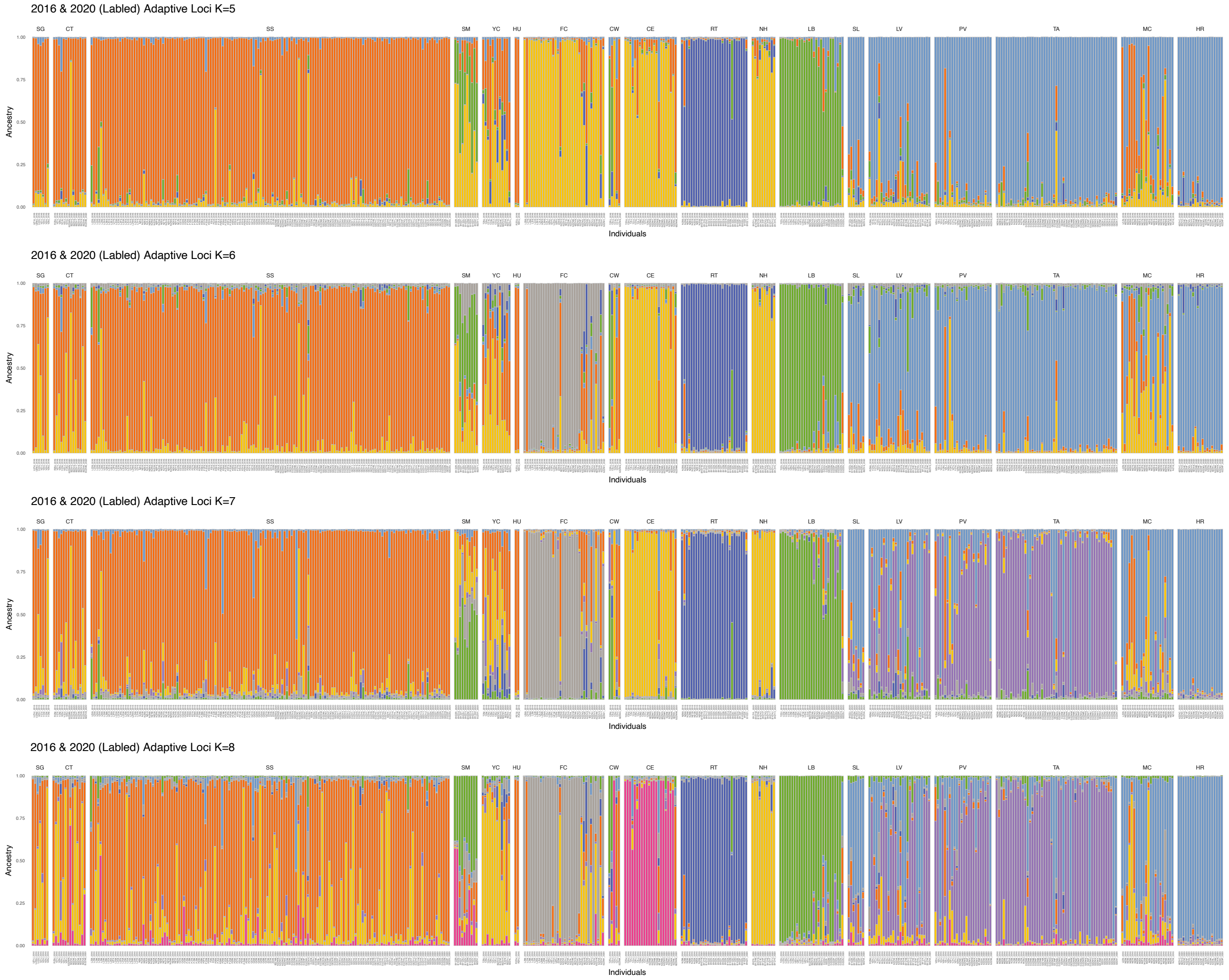

FIGURE S6: PC axes 1-4 for (a,b) all loci, (c,d) neutral loci, and (e,f) adaptive loci with all NIDGS samples plus the disjunct population of RV, represented with one sample by a hot-pink square. Circle points denote the sample was collected in 2016 and triangles for 2020. Colors represent each site

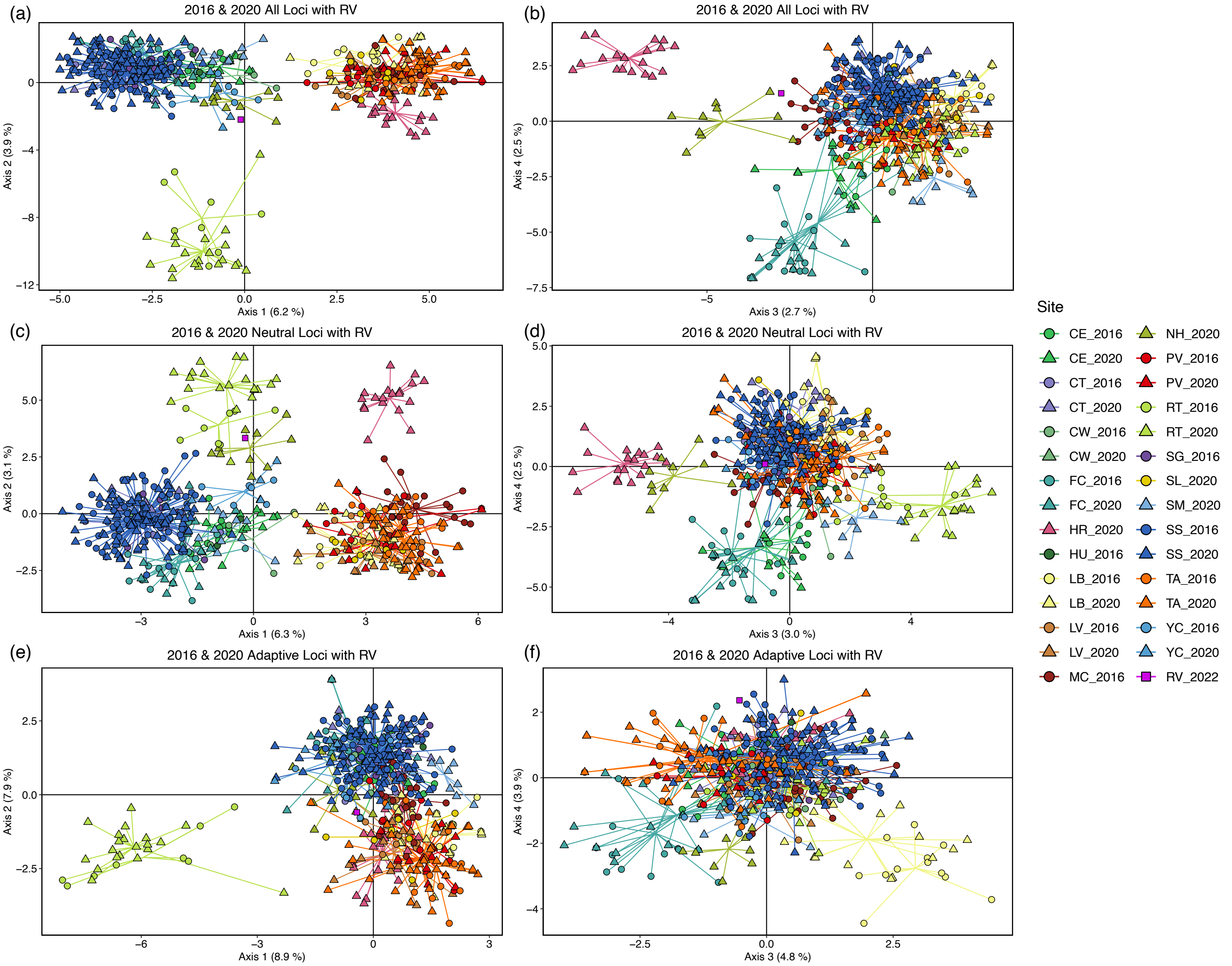

FIGURE S7: Pairwise F_{ST} heatmaps; (a) with all SNPs for 2016 samples, blue represents F_{ST} values of 0 and red represents F_{ST} values of 0.2; (b) with all SNPs for 2020 samples, blue represents F_{ST} values of 0 and red represents F_{ST} values of 0.3; (c) with adaptive SNPs for 2016 samples, blue represents F_{ST} values of 0 and red represents F_{ST} values of 0.3; (d) with adaptive SNPs for 2020 samples, blue represents F_{ST} values of 0 and red represents F_{ST} values of 0.3.

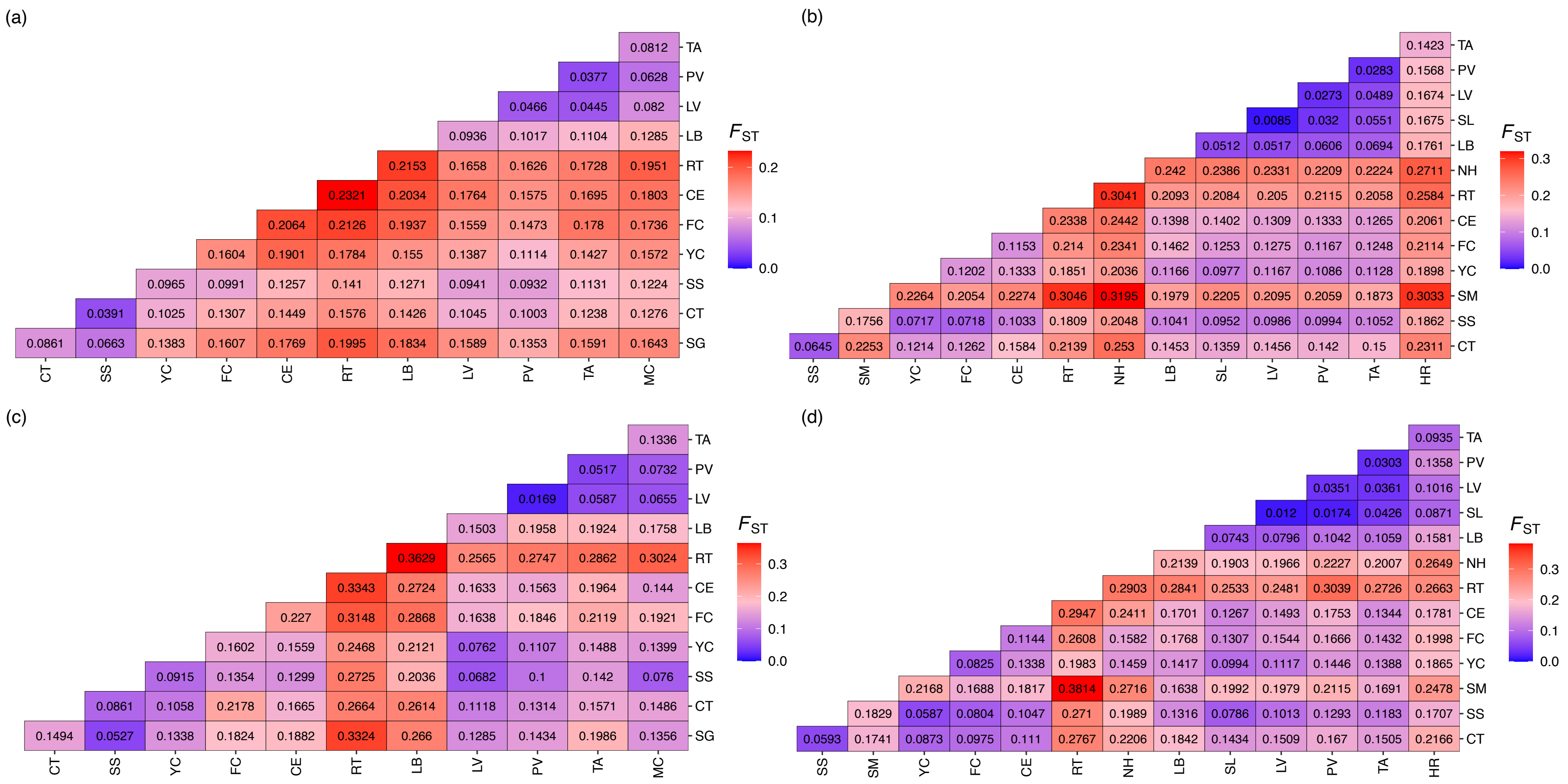

TABLE S1: Mantel test results for IBD in NIDGS for all, neutral and adaptive SNPs by year. IBD was tested with both F_{ST} and Nei's *D*. Bolded values are significant at P ≤ 0.05.

		All SNPs				Neutral SNPs				Adaptive SNPs			
		2016		2020		2016		2020		2016		2020	
		r	p-value	r	p-value	r	p-value	r	p-value	r	p-value	r	p-value
F_{st}	Pearson	0.202	0.077	**0.387**	**0.013**	**0.315**	**0.009**	**0.398**	**0.008**	-0.029	0.575	**0.317**	**0.035**
	Pearson (log of geodist)	**0.337**	**0.001**	**0.406**	**0.001**	**0.4252**	**0.002**	**0.4077**	**0.002**	0.0949	0.164	**0.355**	**0.008**
	Spearman	**0.224**	**0.034**	**0.361**	**0.012**	**0.3386**	**0.007**	**0.3709**	**0.007**	0.0458	0.34	**0.361**	**0.013**
Nei's *D*	Pearson	0.230	0.053	**0.405**	**0.006**	**0.441**	**0.003**	**0.455**	**0.003**	-0.113	0.76	0.137	0.18
	Pearson (log of geodist)	**0.349**	**0.002**	**0.428**	**0.001**	**0.5087**	**0.001**	**0.4526**	**0.001**	0.0111	0.404	**0.2097**	**0.08**
	Spearman	**0.259**	**0.02**	**0.3871**	**0.005**	**0.463**	**0.001**	**0.4289**	**0.005**	0.0399	0.373	**0.3649**	**0.012**

TABLE S2: Frequency of private alleles per locus identified per ESU per year calculated with all loci; * denotes putatively adaptive loci.

Year	ESU	Locus										
		23409246*	2010876*	6382977*	2624181*	3237115	1130422	1561920*	129587	103038	2750	681
2016	West	1	0	0	0	3	0	0	0	-	22	0
	East	0	9	0	22	0	12	0	14	-	0	32
	RT	0	0	7	0	0	0	5	0	-	0	0
2020	West	-	0	-	-	-	0	0	0	43	26	-
	East	-	15	-	-	-	13	0	13	0	0	-
	RT	-	0	-	-	-	0	7	0	0	0	-

TABLE S3: Pairwise F_{ST} confidence intervals for (a) 2016 and (b) 2020 calculated with neutral SNPs only.

(a) **2016 - Neutral Loci**

	CE	CT	FC	LB	LV	MC	PV	RT	SG	SS	TA	YC
**CE**		0.181	0.232	0.220	0.222	0.211	0.190	0.225	0.233	0.143	0.109	0.239
**CT**	0.101		0.159	0.141	0.143	0.156	0.129	0.159	0.110	0.043	0.149	0.134
**FC**	0.162	0.075		0.206	0.197	0.202	0.181	0.209	0.204	0.107	0.206	0.207
**LB**	0.139	0.081	0.169		0.104	0.144	0.105	0.180	0.204	0.123	0.108	0.171
**LV**	0.136	0.068	0.122	0.058		0.114	0.081	0.164	0.212	0.124	0.060	0.189
**MC**	0.144	0.893	0.136	0.083	0.063		0.088	0.181	0.204	0.156	0.087	0.185
**PV**	0.125	0.057	0.103	0.048	0.034	0.037		0.144	0.177	0.112	0.053	0.141
**RT**	0.150	0.085	0.130	0.115	0.105	0.118	0.088		0.192	0.102	0.151	0.183
**SG**	0.122	0.043	0.113	0.122	0.122	0.126	0.094	0.106		0.090	0.183	0.187
**SS**	0.083	0.014	0.061	0.083	0.074	0.108	0.070	0.061	0.045		0.130	0.110
**TA**	0.127	0.078	0.130	0.061	0.023	0.049	0.017	0.089	0.107	0.083		0.167
**YC**	0.153	0.066	0.115	0.101	0.111	0.118	0.074	0.111	0.090	0.065	0.095	

(b) **2020 - Neutral Loci**

	CE	CT	CW	FC	HR	LB	LV	NH	PV	RT	SL	SM	SS	TA	YC
**CE**		0.192	0.182	0.135	0.240	0.165	0.155	0.279	0.162	0.227	0.173	0.260	0.119	0.143	0.165
**CT**	0.127		0.210	0.155	0.271	0.171	0.173	0.299	0.171	0.253	0.171	0.270	0.078	0.169	0.163
**CW**	0.077	0.081		0.179	0.271	0.181	0.170	0.354	0.177	0.275	0.177	0.247	0.050	0.135	0.132
**FC**	0.095	0.100	0.065		0.254	0.175	0.152	0.269	0.138	0.248	0.156	0.232	0.085	0.141	0.150
**HR**	0.174	0.192	0.138	0.175		0.209	0.200	0.308	0.191	0.299	0.203	0.339	0.213	0.166	0.239
**LB**	0.113	0.115	0.064	0.121	0.149		0.066	0.269	0.077	0.244	0.067	0.230	0.124	0.085	0.140
**LV**	0.104	0.114	0.062	0.103	0.139	0.037		0.265	0.040	0.242	0.017	0.234	0.117	0.059	0.143
**NH**	0.206	0.212	0.212	0.189	0.227	0.204	0.192		0.251	0.343	0.284	0.368	0.218	0.236	0.251
**PV**	0.108	0.108	0.057	0.098	0.130	0.043	0.016	0.181		0.247	0.047	0.237	0.118	0.038	0.130
**RT**	0.203	0.181	0.157	0.183	0.220	0.180	0.177	0.256	0.183		0.247	0.341	0.204	0.231	0.228
**SL**	0.110	0.108	0.060	0.099	0.139	0.034	-0.002	0.205	0.015	0.180		0.258	0.116	0.069	0.125
**SM**	0.188	0.186	0.117	0.168	0.253	0.159	0.170	0.271	0.165	0.256	0.185		0.188	0.198	0.276
**SS**	0.080	0.048	-0.034	0.052	0.153	0.088	0.081	0.164	0.082	0.151	0.074	0.133		0.124	0.090
**TA**	0.099	0.114	0.027	0.102	0.111	0.053	0.036	0.177	0.018	0.171	0.035	0.143	0.090		0.131
**YC**	0.105	0.079	0.011	0.091	0.155	0.091	0.086	0.169	0.085	0.153	0.069	0.194	0.055	0.087	

TABLE S4: Private alleles per locus by each candidate AU (*K*=3-5) per year.

Unit		Locus				
		23409246	2010876	6382977	2624181	1561920
**K3 - 2016**	West	1	0	0	0	0
	East	0	9	0	22	0
	RT	0	0	7	0	5
**K4 - 2016**	Central	0	0	0	0	0
	West	1	0	0	0	0
	East	0	9	0	22	0
	RT	0	0	7	0	5
**K5 - 2016**	Central	0	0	0	NA	0
	West	1	0	0	NA	0
	LB	0	0	0	NA	0
	East	0	9	0	NA	0
	RT	0	0	7	NA	5
**K3 - 2020**	West	NA	0	NA	NA	0
	East	NA	15	NA	NA	0
	RT	NA	0	NA	NA	7
**K4 - 2020**	Central	NA	0	NA	NA	0
	West	NA	0	NA	NA	0
	East	NA	15	NA	NA	0
	RT	NA	0	NA	NA	7
**K5 - 2020**	Central	NA	0	NA	NA	0
	West	NA	0	NA	NA	0
	LB	NA	0	NA	NA	0
	East	NA	15	NA	NA	0
	RT	NA	0	NA	NA	7
